# Dopamine D1 and D2 Receptors Are Important for Learning About Neutral-Valence Relationships in Sensory Preconditioning

**DOI:** 10.3389/fnbeh.2021.740992

**Published:** 2021-08-30

**Authors:** Stephanie Roughley, Abigail Marcus, Simon Killcross

**Affiliations:** School of Psychology, UNSW Sydney, Sydney, NSW, Australia

**Keywords:** dopamine, preconditioning, pavlovian, learning, D1, D2

## Abstract

Dopamine neurotransmission has been ascribed multiple functions with respect to both motivational and associative processes in reward-based learning, though these have proven difficult to tease apart. In order to better describe the role of dopamine in associative learning, this series of experiments examined the potential of dopamine D1- and D2-receptor antagonism (or combined antagonism) to influence the ability of rats to learn neutral valence stimulus-stimulus associations. Using a sensory preconditioning task, rats were first exposed to pairings of two neutral stimuli (S2-S1). Subsequently, S1 was paired with a mild foot-shock and resulting fear to both S1 (directly conditioned) and S2 (preconditioned) was examined. Initial experiments demonstrated the validity of the procedure in that measures of sensory preconditioning were shown to be contingent on pairings of the two sensory stimuli. Subsequent experiments indicated that systemic administration of dopamine D1- or D2-receptor antagonists attenuated learning when administered prior to S2-S1 pairings. However, the administration of a more generic D1R/D2R antagonist was without effect. These effects remained constant regardless of the affective valence of the conditioning environment and did not differ between male and female rats. The results are discussed in the context of recent suggestions that dopaminergic systems encode more than a simple reward prediction error, and provide potential avenues for future investigation.

## Introduction

Dopamine neurotransmission has been shown to be critical for aspects of reward-related learning in many different preparations, making it of key interest in the study of disorders involving dysregulation of the reward system, such as addiction, depression, ADHD, and schizophrenia. However, it has proven difficult to pinpoint the precise function(s) of dopamine with respect to associative learning (the formation of connections between cues or actions and their associated outcomes) and motivational processes. Whilst there is strong background literature implicating dopamine in aspects of effort (Salamone et al., 2007, 2016; Salamone and Correa, 2012), desire (Berridge, 2007; Flagel et al., 2011) and reward (Wise, 2006, 2008), there is also substantial evidence highlighting its importance in the prediction error mechanisms important for associative formation in appetitive Pavlovian conditioning and instrumental reinforcement learning (Schultz et al., 1997; Schultz and Dickinson, 2000; Schultz, 2007; Balleine et al., 2009; Wassum et al., 2012; Steinberg et al., 2013; Chang et al., 2016).

Although it seems very likely that dopamine in fact plays a role in most (if not all) of these processes, it is not straightforward to isolate the different neurochemical mechanisms, and their neuroanatomical loci, through which dopamine is able to perform these different functions. One reason for this is that the majority of work investigating these functions naturally takes place in the context of reward-based conditioning procedures, making it difficult to tease apart the separate processes involved and study them in isolation. Accordingly, in order to get a clear picture of dopamine’s role in learning, it may help to look beyond reward-learning procedures such as appetitive conditioning and reinforcement learning and explore dopamine’s involvement in other associative preparations. For example, there is evidence to suggest that dopamine may be important in neutral-valence, stimulus-stimulus learning (Young et al., 1998; Sharpe et al., 2017).

One protocol used to examine this type of learning is sensory preconditioning (e.g., Holmes et al., 2013). In the first stage of a sensory preconditioning procedure, animals are exposed to pairings between two innocuous sensory cues (S2-S1; e.g., a tone and a light). In Stage 2, S1 is then paired with a mildly aversive unconditioned stimulus (US), such as a foot-shock (S1-US; e.g., light-shock). The degree to which animals learned about the S2-S1 relationship can then be assessed by measuring fear expressed to S2 (which was never directly paired with the fear-inducing US); Animals learn to fear S1 *via* S1-US conditioning, and as a consequence of having already learned the S2-S1 relationship, come to fear S2 by association. This procedure provides an opportunity to study the neural mechanisms involved in the formation of associations between stimuli that, at the time of learning, have no motivational significance—thereby separating the associative learning process from potentially confounding motivational functions. In addition, the nature of the stimuli and patterns of presentation are more closely matched to previous protocols demonstrating an impact of dopaminergic manipulation than other commonly employed sensory preconditioning procedures such as those involving flavour-flavour associations.

The basic notion that dopamine may be involved in learning about sensory stimuli is evidenced by early work demonstrating dopamine neurons fire in response to novel or high-intensity stimuli, or unexpected stimuli of a sort capable of eliciting a behavioural response (e.g., orienting) before these are ever paired with reward (Schultz and Romo, 1990; Ljungberg et al., 1992; Horvitz, 2000). In the context of sensory preconditioning, dopamine levels have been shown to increase in the nucleus accumbens during sensory S2-S1 learning (but not unpaired S2/S1 presentations) and subsequent tests of both S2 and S1 (Young et al., 1998). Similarly, dopamine neurons in the ventral tegmental area have been observed to fire in response to preconditioned sensory cues never directly paired with reward (Sadacca et al., 2016) and activation of these neurons has been shown to be necessary and sufficient to drive S2-S1 learning (Sharpe et al., 2017).

These studies demonstrate that dopamine is involved in learning processes that occur in the absence of any explicit reward factor, which has important implications for our broader understanding of dopamine function. However, much remains to be explored with regards to the nature of dopamine’s involvement in this learning. For example, the evidence to date primarily stems from studies investigating dopamine release and/or activity in dopaminergic neurons. Little is known about downstream mechanisms involving activity at specific dopamine receptor subtypes or the neural populations and circuits in which these are expressed.

Evidence from the appetitive literature highlights that distinct dopamine receptor subtypes can serve complementary functions in some situations, and competing functions in others. For example, some have found evidence of D1R- and D2R-activation working in concert (Capper-Loup et al., 2002; Perreault et al., 2014; Kupchik et al., 2015; Hasbi et al., 2018) In contrast, it has been shown that Pavlovian cued food approach is impaired by D1R antagonism but enhanced by D2R antagonism (Eyny and Horvitz, 2003). Similarly, in an instrumental paradigm, administration of amphetamine promotes the development of habitual behaviour and this effect is prevented by blockade of D1R but enhanced by blockade of D2R (Nelson and Killcross, 2006, 2013).

Furthermore, we have shown that the acquisition of anticipatory approach behaviour towards the location of predicted reward delivery in an appetitive Pavlovian conditioning procedure requires activity at dopamine D1-like receptors (D1R) but not D2-like receptors (D2R; Roughley and Killcross, 2019). In contrast, acquisition of approach behaviour towards a *cue* that predicts reward delivery requires activity at both D1R and D2R (Roughley and Killcross, 2019). These findings are broadly consistent with other evidence that appears to suggest that D1R, and the phasic firing pattern of dopamine neurons for which these receptors have a preferential affinity (Wall et al., 2011), might be particularly important for learning predictive relationships between contingent events in general (Schultz, 2007; Zweifel et al., 2009), whereas D2R, more sensitive to tonic dopamine release resulting from basal level firing, may be more selectively involved in motivational aspects of learning and performance (Niv, 2007; Salamone and Correa, 2012; Gallo, 2019). In light of the substantial similarity between D1- and D5-receptors and D2- and D3/D4-receptors (including with respect to the specificity of dopaminergic agonists/antagonists), it should be noted that whilst we refer in this article to D1- and D2-receptors, these terms relate to D1-like and D2-like receptor families more broadly.

It would be of interest to explore the potentially differential or cooperative role of D1R and D2R in the context of sensory stimulus-stimulus learning. Accordingly, the aim of the present set of experiments is to determine whether the activity at D1R and/or D2R is important for the acquisition of S2-S1 associations in a sensory preconditioning procedure. In the interest of comparison with appetitive conditioning procedures, a further aim is to investigate whether the nature of any D1R/D2R involvement is influenced by the motivational context in which learning takes place. In this way, we hope to demonstrate the utility of higher-order conditioning procedures like sensory preconditioning in enhancing our understanding of the neurochemical mechanisms at play in psychological disorders, such as addiction, that involve dysregulated associative learning processes.

In Experiment 1 we provide a demonstration of sensory preconditioning in a neutral vs. motivationally attractive context and confirm that, in both cases, the fear expressed to S2 at test is specifically a function of learned associations between S2-S1 and S1-US, and not due to generalisation effects or inherent conditioning properties of the stimuli (Holmes et al., 2013). Experiment 2 examines the importance of D1R and D2R for S2-S1 learning through the systemic administration of selective D1R- or D2R-antagonists prior to Stage 1 of the sensory preconditioning procedure. Experiment 3 also investigates the role of D1R and D2R in S2-S1 learning, but in this case it takes place in an environment already established as attractive. Finally, in Experiment 4 we examine whether the effects of D1R and D2R antagonism on S2-S1 learning are additive, and again, whether this differs according to the motivational relevance of the learning environment.

## Materials and Methods

### Subjects

Subjects were experimentally naïve, male and female Long-Evans rats (UNSW Psychology breeding colony), between 12–16 weeks of age at the beginning of experimental procedures. Rats were housed in groups of four, in a temperature- and humidity-controlled environment (22°C) operating on a 12 h light/dark cycle (lights on at 07:00 h). Experimental procedures took place during the light cycle. For Experiments 1, 3, and 4 rats were placed on a restricted food schedule prior to behavioural training to induce appetitive motivation for food. Weights never reduced past 85% of free-feeding values and water was continuously available in home-cages. In Experiment 2 both food and water were continuously available. Animal procedures were carried out in accordance with the National Institute of Health Guide for the Care and Use of Laboratory Animals (NIH publications No. 80–23, revised 1996), and were approved by the UNSW Animal Care and Ethics Committee.

### Apparatus

Behavioural training and testing took place in eight standard operant chambers (30 cm × 24 cm × 22 cm; MED Associates Inc., St. Albans, VT, USA), each housed in a light- and sound-attenuating compartment. The sidewalls of each chamber were constructed of aluminium, and the back wall, ceiling, and hinged front wall were made of clear Perspex. Floors consisted of 19 stainless-steel bars (4 mm diameter; 1.5 cm apart), aligned perpendicular to the back wall of the chamber. A constant current shock generator (MED Associates Inc., St. Albans, VT, USA) was used to deliver a brief duration electric current to the grid floor of the chamber (0.8 mA intensity; 0.5 s duration). Floors were cleaned after each experimental session.

The auditory stimulus used was a 70 dB 1 kHz square-wave tone produced through a speaker located at the top back left corner of the chamber. The visual stimulus was a 28 W light located on the ceiling of the compartment, flashing at a rate of approximately 3 Hz. The physical identity of the stimuli was fully counterbalanced in each experiment. Chambers were also equipped with a recessed food magazine located at the bottom Centre of the right-hand wall, into which reward pellets could be delivered from a pellet dispenser (Experiments 1, 3, and 4). Head entries to the magazine were detected by breaks of an infrared beam across the opening of the magazine.

Each chamber was illuminated *via* an infrared light source on the compartment ceiling. Cameras mounted on the back wall of each compartment recorded rats’ behaviour during training and test sessions. Recordings were stored on an external hard drive. Experimental events were controlled and recorded *via* a PC running Med-PC software.

### Drugs

Dopamine receptor antagonists were dissolved in 0.9% saline (w/v) and injected subcutaneously at a volume of 1 ml/kg 15 min prior to sensory preconditioning (Stage 1 training session). For Experiments 2 and 3, the antagonists used were the selective D1R antagonist SCH39166 (Tocris Bioscience; Bristol, UK), administered at a dose of 0.0125 mg/kg (Low), 0.025 mg/kg (Mid), or 0.05 mg/kg (High), and the selective D2R antagonist eticlopride hydrochloride (Sigma-Aldrich; Sydney, Australia), administered at a dose of 0.003 mg/kg (Low), 0.0125 mg/kg (Mid), or 0.03 mg/kg (High). For Experiment 4, the antagonist used was the non-selective dopamine receptor antagonist α-flupenthixol (flupenthixol dihydrochloride; Sapphire Bioscience; Redfern, Australia), administered at a dose of 0.5 mg/kg. SCH39166 and eticlopride doses were determined on the basis of the range observed within our laboratory to be behaviourally effective in an appetitive conditioning context (e.g., Roughley, 2017; Roughley and Killcross, 2019). The dose of α-flupenthixol was deliberately chosen to be at the high end of that range; at this dose, motor function remains intact but animals show much reduced performance of motivated behaviours (e.g., Roughley and Killcross, 2019). In all experiments, the vehicle solution for control injections was physiological saline (0.9% w/v).

### Behavioural Procedures

#### Pretraining

Rats were handled daily in the week preceding the onset of experimental procedures to familiarise them with the experimenter and basic protocols. In Experiments 1, 3, and 4, on two days prior to the start of training, rats were familiarised with the food pellets they would receive during context exposure (45 mg grain pellets; Bio-Serve, Frenchtown, NJ, USA).

#### Context Exposure

**Figure 1:**
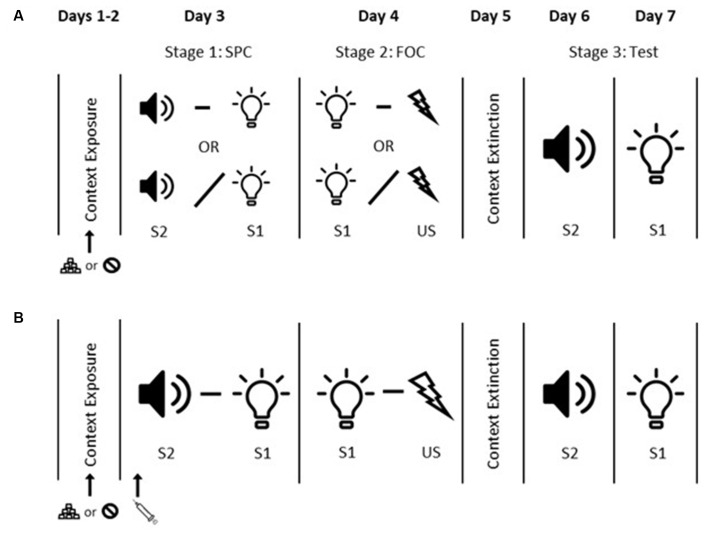
Sensory preconditioning experimental timeline. Panel **(A)** shows the timeline for Experiment 1. Days 1 and 2 = context exposure. On Day 2 rats in the Appetitive condition received food pellet exposure during context exposure while rats in the Neutral condition did not. Day 3 = sensory preconditioning (SPC). Rats in Groups P-P and P-UP received paired S2 and S1 presentations (tone and light; counterbalanced). S2 and S1 were presented in an unpaired fashion for Group UP-P. Day 4 = first-order conditioning (FOC). Rats in Groups P-P and UP-P received paired S1-US (shock) presentations. S1 and US were presented in an unpaired fashion for Group P-UP. Day 5 = context extinction. Days 6 and 7 = tests of S2 alone and S1 alone. Panel **(B)** shows the timeline for Experiments 2–4. Days 1 and 2 = context exposure. On Day 2 all rats in Experiment 3 and rats in the Appetitive condition in Experiment 4 received food pellet exposure during context exposure. Rats in Experiment 2 and rats in the Neutral condition in Experiment 4 did not. Day 3 = SPC. All rats received paired S2-S1 presentations. Injections of dopamine antagonist or vehicle (according to the group) were administered prior to the SPC session. Day 4 = FOC. All rats received paired S1-US presentations. Day 5 = context extinction. Days 6 and 7 tests of S2 alone and S1 alone.

A summary of the experimental timeline from context exposure onwards can be found in (Figure 1). Experimental protocols are based on those used by Holmes and colleagues (e.g., Holmes et al., 2013). On each of the first two days of the experimental protocol, rats received two 30-min sessions of exposure to the conditioning chamber. These were separated by a minimum interval of 3 h. For experiments/groups in which sensory preconditioning took place in a neutral context (1, 2, 4), no programmed events took place during exposure sessions. In other experiments (1, 3, 4), in order to establish the conditioning chamber as an appetitive context, food pellets were delivered periodically to the food magazine in the chamber throughout the final two exposure sessions (variable time 60 s; approximately one pellet per minute). In all cases rats reliably came to retrieve pellets from the magazine. These were the only sessions in which rats received any food in the chambers.

#### Sensory Preconditioning

Stage 1 sensory preconditioning (SPC) occurred over a single session approximately 46 min in duration. The session involved eight presentations each of the visual and auditory stimuli, designated S1 or S2 (fully counterbalanced). S2 was presented on a fixed time schedule with 5 min 10 s between each presentation, and each presentation lasting 30 s. In groups receiving paired (contingent) S2-S1 presentations, S1 began at the termination of S2. S1 presentations lasted 10 s. In groups receiving unpaired (non-contingent) S2 and S1 presentations, S1 was presented in the middle of the interval between S2 presentations. Rats remained in the conditioning chamber for 1 m following the final stimulus presentation. For experiments involving drug manipulations (Experiments 2, 3 and 4), animals were randomly assigned to receive an injection of either saline or dopamine antagonist prior to this session.

#### First-Order Conditioning

Stage 2 first-order fear conditioning (FOC) took place the next day over a single session of approximately 22 min duration. The session involved four 10 s presentations of the stimulus previously designated S1 (either tone or light, counterbalanced) and four presentations of the US (a 0.5-s, 0.8-mA foot-shock). S1 presentations were separated by an interval of 5 min. For groups receiving paired S1-US presentations, the shock was delivered in the final 0.5 s of each S1 presentation. For groups receiving unpaired S1 and US presentations, the shock was delivered in the middle of the interval between S1 presentations. Rats remained in the conditioning chamber for 1 min following the final stimulus presentation.

#### Context Extinction

On the day following FOC, rats received two 30-min sessions of context extinction in which they were placed in the conditioning chamber but no programmed events occurred. These sessions were separated by a minimum interval of 3 h. The purpose of these sessions was to extinguish any freezing elicited by the context, in order to better observe freezing specifically elicited by conditioned stimuli on subsequent tests.

#### Testing

On test days rats first received an additional 10 min of context extinction, followed 2 h later by the test session. Test sessions involved 8 presentations of the S2 stimulus alone (Test 1) or S1 stimulus alone (Test 2; 24 h later). In each test stimulus durations were as in training (S2 = 30 s; S1 = 10 s) and presentations were separated by 3-min intervals.

### Data Analysis

Freezing was the measure of conditioned fear, defined as the absence of all movement (except breathing) in an awake animal. For each rat, observations were made every 2 s during stimulus presentations and a baseline period (2 min at the start of session), where the rat was scored as either “freezing” or “not freezing.” Scoring was conducted by an observer blind to experimental condition. A proportion of observations (~10%) were cross-scored by a second independent observer (also blind to experimental conditions) to ensure observer reliability. In all cases, there was a high degree of agreement between primary observer and cross-scorer (<90%). Overall freezing scores were expressed as a percentage of total observations and used to calculate a difference score (% freezing during stimulus presentations − % freezing during baseline) to be used in statistical analysis. Data were analysed using between-subjects univariate analysis of variance (ANOVA) with *post hoc* pairwise comparisons (Scheffe correction for multiple comparisons) where relevant. Significance was set at *α* = 0.05.

## Results

### Experiment 1: Sensory Preconditioned Fear in a Neutral vs. Appetitive Context

Experiment 1 comprised a control study based on Holmes et al. (2013), designed to demonstrate that fear of S2 at test (indexed by freezing) is a function of the learned associations between both S2 and S1, and S1 and shock, and that changing the valence of the context (neutral vs. appetitive) does not in and of itself change the basic associative processes in sensory preconditioning.

Forty-eight rats were randomly allocated to receive training in either a neutral or appetitive context. For the Appetitive condition, rats were placed on a food restriction schedule during handling. Context exposure occurred on Days 1 and 2, and for the Appetitive condition Day 2 exposure involved the delivery of food rewards throughout both sessions in order to establish the training context as a positive environment. This manipulation was shown to be successful, as indicated by a significant increase in entries to the food magazine per min from the first to the second session of context exposure with food presentation [*t*-test; *t*_(23)_ = 3.989, *p* = 0.001, where mean (±SD) for session 1 = 9.919 (±3.834) and session 2 = 12.892 (±4.629)]. For the Neutral condition, context exposure proceeded without any programmed events.

Rats in each condition were randomly divided into three further groups (*n* = 8; 4M and 4F) that received either paired or unpaired stimulus presentations during SPC and/or FOC sessions. In the experimental group, Group P-P (paired-paired condition), a contingent relationship was established between both S2-S1 and S1-US; rats received contingent S2-S1 presentations during the SPC session and contingent S1-Shock presentations during the FOC session. In the control groups, Group P-UP (paired-unpaired condition) and Group UP-P (unpaired-paired condition), a contingent relationship was established for only one of the stimulus pairs (either S2-S1 or S1-US). Group P-UP received paired S2-S1 presentations during the SPC session, but explicitly unpaired S1 and shock presentations during the FOC session. In reverse, Group UP-P received explicitly unpaired S2 and S1 presentations in the SPC session and paired S1-Shock presentations during the FOC session. Context extinction was carried out for all rats on Day 5, and tests of freezing to S2 and S1 were conducted on days 6 and 7, respectively.

**Figure 2:**
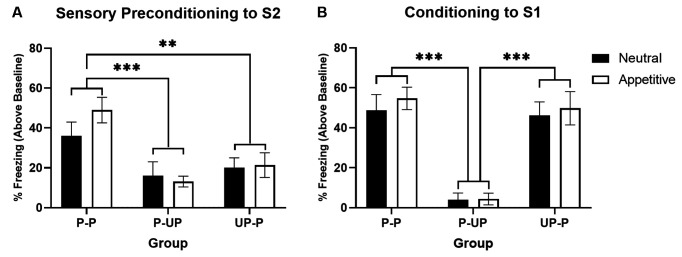
Sensory preconditioning in a neutral vs. appetitive context. Panel **(A)** shows the average per cent time spent freezing in response to S2 (stimulus—baseline) on the test of sensory preconditioning when groups were trained in a neutral context vs. appetitive context. Groups P-P show significantly more freezing than either Groups P-UP or UP-P, irrespective of context valence. Panel **(B)** shows the average per cent time spent freezing to S1 (stimulus—baseline) on the test of first-order conditioning when groups were trained in a neutral context vs. appetitive context. Groups P-UP show significantly less freezing than either Groups P-P or UP-P, irrespective of context valence. Black bars indicate groups trained in a neutral context, while white bars indicate groups trained in an appetitive context (*n* = 8 for all groups). Groups P-P experienced paired S2-S1 and S1-US training; Groups P-UP experienced paired S2-S1 and unpaired S1 and US training; Groups UP-P experienced unpaired S2 and S1 and paired S1-US training. Error bars represent ±SEM. Significant comparisons are indicated by: ** where *p* < 0.01; *** where *p* < 0.001.

Figure 2A shows average levels of freezing in response to S2 during the test for the Neutral context and Appetitive context conditions (stimulus-baseline; averaged across eight S2 presentations). In both Neutral and Appetitive conditions, freezing was significantly higher in Group P-P than either Group P-UP or UP-P. A two-way ANOVA with between subject factors of Group (P-P, P-UP, and UP-P) and Context (Appetitive and Neutral) revealed a significant main effect of Group (*F*_(2,42)_ = 12.465; *p* < 0.001) but no main effect of Context or Group by Context interaction (both *F* < 1). *post hoc* pairwise comparisons indicate that averaging across context, freezing was significantly higher in Groups P-P than either P-UP (*p* < 0.001) or UP-P (*p* = 0.003).

Figure 2B shows average levels of freezing to S1 during the test for the Neutral and Appetitive conditions. Freezing was higher in the P-P and UP-P groups than the P-UP groups, irrespective of context condition. A two-way ANOVA revealed a significant main effect of Group (*F*_(2,42)_ = 37.126; *p* < 0.001) but no main effect of Context or Group by Context interaction (both *F* < 1). *Post hoc* pairwise comparisons indicate that averaging across context, freezing was significantly higher in Groups P-P and UP-P compared to P-UP (both *p* < 0.001).

These results demonstrate that freezing to S2 was dependent on its contingent relationship to S1 and also the contingent relationship between S1 and shock. Furthermore, this did not differ as a function of context valence. Thus, whether SPC occurred in a Neutral or Appetitive context, fear to S2 reflected learned associations between S2 and S1, and S1 and shock, rather than being a function of an inherent ability of S1 to condition fear (which would be indicated by high responding in Group P-UP) or of fear generalisation to S2 from S1 (which would be indicated by high responding in Group UP-P).

### Experiment 2: Blocking Dopamine D1 or D2 Receptors Impairs Learning of Neutral S2-S1 Associations

The aim of Experiment 2 was to investigate dopamine’s involvement in learning associations between neutral stimuli. Specifically, we aimed to determine whether systemic blockade of activity at dopamine D1R and/or D2R impaired S2-S1 learning during a sensory preconditioning procedure, as indexed by subsequent impairments in freezing to S2 at test. In addition, to further the broader aim of improving the generalisability of findings in behavioural neuroscience, male and female rats were used in this experiment and sex was assessed as a factor.

All rats underwent exposure sessions on Days 1 and 2 in a neutral context. Immediately prior to the SPC session, rats were randomly allocated to receive an injection of either saline (Group VEH), one of three doses of the D1R antagonist SCH39166 (Groups SCH-Low, SCH-Mid and SCH-High), or one of three doses of the D2R antagonist eticlopride (ETI-Low, ETI-Mid, or ETI-High). In this experiment, all rats received paired S2-S1 presentations during the SPC session on Day 3 and paired S1-shock presentations during the FOC session on Day 4. Context extinction was conducted on Day 5, and tests of S2 and S1 were carried out on Days 6 and 7, respectively.

Due to a camera malfunction, behaviour was not recorded during the test of S1 for one rat and so it was excluded from analysis. Final group sizes (*N* = 91) were as follows: VEH, *n* = 16 (8F; 8M); SCH-Low, *n* = 14 (7F; 7M); SCH-Mid, *n* = 12 (6F; 6M); SCH-High, *n* = 13 (6F; 7M); ETI-Low, *n* = 12 (6F; 6M); ETI-Mid, *n* = 12 (6F; 6M); ETI-High, *n* = 12 (6F; 6M).

**Figure 3:**
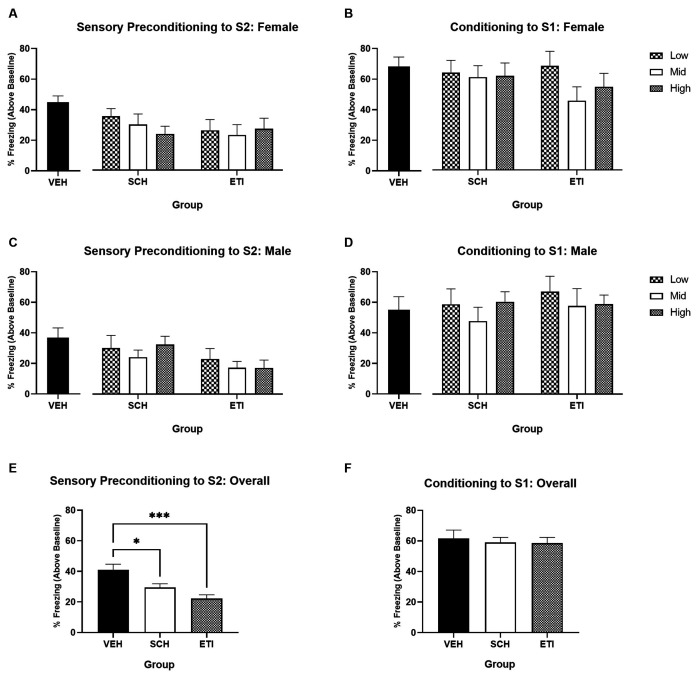
Sensory preconditioning under conditions of dopamine receptor antagonism in male and female rats. Training in a neutral context. Panels **(A,C)** show average per cent time spent freezing in response to S2 (stimulus—baseline) on the test of sensory preconditioning in groups of female vs. male rats, respectively. Panels **(B,D)** show average per cent time spent freezing to S1 (stimulus—baseline) on the test of first-order conditioning in groups of female vs. male rats, respectively. In Panels **(A–D)**, black bars indicate treatment with saline vehicle, black and white chequered bars indicate treatment with the low-dose antagonist, white bars indicate treatment with the mid-dose antagonist, and grey hashed bars indicate treatment with the high-dose antagonist. Panels **(E,F)** show the average per cent time freezing (stimulus—baseline) during tests of S2 and S1 respectively, collapsed across dose and sex. Vehicle-treated rats (VEH; *n* = 16) froze significantly more than SCH39166- (SCH; *n* = 39) or eticlopride-treated rats (ETI; *n* = 36). Error bars represent ±SEM. Significant comparisons are indicated by: * where *p* < 0.05; *** where *p* < 0.001.

Figures 3A,C show the average levels of freezing in response to S2 during the test in Female and Male rats, respectively (stimulus-baseline; averaged across eight S2 presentations). In both male and female rats, freezing to S2 was significantly higher for those in the vehicle-treated group than SCH39166- or eticlopride-treated groups. However, no dose-dependent effect was apparent for either SCH39166 or eticlopride. For illustrative purposes, Figure 3E shows comparisons between VEH, SCH, and ETI conditions collapsed across dose and sex.

A two-way ANOVA with between-subjects factors of Drug Type (VEH, SCH, and ETI; averaging across dose) and Sex (male and female) revealed a significant main effect of Drug Type (*F*_(2,85)_ = 8.802, *p* < 0.001), but no main effect of Sex (*F*_(1,85)_ = 2.160, *p* = 0.145) or Sex by Drug Type interaction (*F* < 1). *post hoc* pairwise comparisons indicate that averaging across sex, freezing was significantly higher in vehicle-treated rats than either SCH39166- (*p* = 0.040) or eticlopride-treated rats (*p* < 0.001). Follow-up two-way ANOVAs with between-subjects factors of Dose (Low, Mid, and High) and Sex (male and female) did not reveal any significant main effects of Dose or Sex, or Dose by Sex interaction in either the SCH groups (all *F* < 1) or ETI groups (*F* < 1; *F*_(1,30)_ = 1.526, *p* = 0.226; *F* < 1, respectively). Together, this indicates that irrespective of dose, blockade of dopamine D1R or D2R impaired learning during sensory preconditioning.

Figures 3B,D show average levels of freezing to S1 during the test in Female and Male rats, respectively. Figure 3F shows comparisons between VEH, SCH, and ETI conditions collapsed across dose and sex. There were no significant drug effects, irrespective of sex. A two-way ANOVA revealed no significant main effect of Drug Type (*F* < 1), Sex (*F* < 1), nor Sex by Drug Type interaction (*F*_(2,85)_ = 1.188, *p* = 0.310). This indicates that the deficits in S2 freezing observed above in drug-treated groups was not a function of impaired conditioning to S1. Together these results demonstrate that activity at dopamine D1 and D2 receptors is important for learning the association between S2 and S1, in both male and female rats.

### Experiment 3: Dopamine D1 vs. D2 Receptor Involvement in Learning S2-S1 Associations in an Appetitive Context

Experiment 2 highlighted dopamine’s importance in the learning of neutral associations between sensory cues, confirming and extending previous work by demonstrating the integral role of activity at both dopamine D1- and D2-receptors. However, although it is not without precedent that D1R and D2R are found to subserve similar or cooperative roles, the failure to find any evidence for differential function in dopamine receptor subtypes in this context was somewhat surprising given the existing literature. Specifically, the hypothesis that it is D1R, but not D2R, that are particularly implicated in the formation of conditioned associations. Accordingly, it is of interest to further investigate the conditions under which D1R and D2R are recruited for learning.

The aim of Experiment 3 was to assess whether differences in D1R vs. D2R involvement may be observed if otherwise neutral S2-S1 learning took place in a motivationally significant environment. The rationale behind this is two-fold. Firstly, most of the related background literature examines dopamine’s role in reward-learning (e.g., appetitive Pavlovian conditioning and reinforcement learning), which always takes place in an appetitive context. Together with Experiment 2, this study will provide a valuable comparison that may be able to shed more light on the precise function(s) of dopamine in the more typical appetitive conditioning preparations.

Secondly, there is existing evidence to indicate that the neural structures and mechanisms recruited for innocuous stimulus-stimulus learning are influenced by the nature of the context in which that learning takes place (Holmes et al., 2013, 2018; Holmes and Westbrook, 2017). For the more general purpose of enhancing our understanding of the underlying processes involved in sensory preconditioning, it is of interest to assess whether the role of dopamine in S2-S1 learning is similarly sensitive to the motivational significance of the context.

Since no differential effects of dose (for either SCH39166 or eticlopride) were observed in Experiment 2, only the mid dose of each antagonist was used in this study (0.025 mg/kg and 0.0125 mg/kg, respectively). Furthermore, since there was no evidence from Experiment 2 that S2-S1 learning, or the impact of dopamine antagonism on S2-S1 learning, differed as a function of sex, male and female rats in this experiment were grouped together.

All rats were placed on a food restriction schedule during handling, and context exposure occurred on Days 1 and 2. Day 2 involved exposure to food rewards throughout both sessions in order to establish the training context as a positive environment. There was a significant increase in entries to the food magazine per min from the first to the second session of context exposure with food presentation [*t*-test; *t*_(35)_ = 4.320, *p* < 0.001, where mean (±SD) for session 1 = 10.848 (±3.551) and session 2 = 14.070 (±4.603)], indicating that the manipulation was successful. Immediately prior to the SPC session, rats were randomly allocated to receive an injection of either saline (Group VEH), SCH39166 (Group SCH), or eticlopride (Group ETI). In this experiment, all rats received paired S2-S1 presentations during the SPC session on Day 3 and paired S1-shock presentations during the FOC session on Day 4. Context extinction was conducted on Day 5, and tests of S2 and S1 were carried out on Days 6 and 7, respectively. One rat did not receive a shock during the FOC session and was excluded from the analysis. Final group sizes (*N* = 35) were as follows: Group VEH, *n* = 11 (5F; 6M); Group SCH, *n* = 12 (6F; 6M); Group ETI, *n* = 12 (6F; 6M).

**Figure 4:**
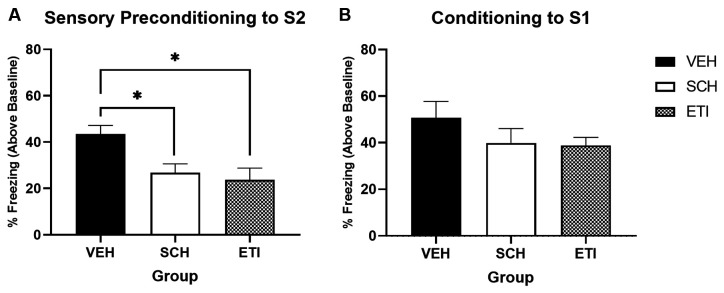
Sensory preconditioning under conditions of dopamine receptor antagonism when trained in an appetitive context. Panel **(A)** shows the average per cent time spent freezing in response to S2 (stimulus—baseline) on the test of sensory preconditioning. Freezing was significantly higher in vehicle-treated rats (VEH; *n* = 11) compared to either SCH39166- (SCH; *n* = 12) or eticlopride-treated rats (ETI; *n* = 12). Panel **(B)** shows the average per cent time spent freezing to S1 (stimulus—baseline) on the test of first-order conditioning. Black bars indicate treatment with saline vehicle, white bars indicate treatment with (mid-dose) D1R antagonist SCH39166, and grey hashed bars indicate treatment with (mid-dose) D2R antagonist eticlopride. Error bars represent ±SEM. Significant comparisons are indicated by: * where *p* < 0.05.

Figure 4A shows the average levels of freezing in response to S2 at the test (stimulus-baseline; averaged across eight S2 presentations). Freezing was significantly impaired in both drug-treated groups relative to the saline-treated group. A one-way univariate ANOVA revealed a significant effect of Drug Type (Saline, SCH, and ETI; *F*_(2,32)_ = 5.957, *p* = 0.006), with *post hoc* pairwise comparisons confirming that freezing in Group Saline was significantly higher than in Group SCH (*p* = 0.035) or Group ETI (*p* = 0.011). Thus, when exposure occurs in an appetitive context, dopamine antagonism at either D1R or D2R impairs learning of the S2-S1 association.

Figure 4B shows the average levels of freezing in response to S1 at test. There was no evidence to suggest responses differed between groups, with a one-way univariate ANOVA revealing no significant effect of Drug Type (Saline, SCH, and ETI; *F*_(2,32)_ = 1.287, *p* = 0.290). This shows that the deficits in S2 freezing observed in drug-treated groups was not a function of impaired conditioning to S1. These results complement those of Experiment 2, demonstrating that when sensory preconditioning occurs in a motivationally attractive context (just as for when it occurs in a neutral context) activity at dopamine D1 and D2 receptors is important for learning an association between S2 and S1.

### Experiment 4: Blocking Dopamine D1 and D2 Receptors Together Does Not Appear to Impact Learning of S2-S1 Associations

Results of Experiment 3 further confirm those of Experiment 2, demonstrating that both D1R and D2R activity is important in the learning of neutral-stimulus S2-S1 associations. Together with Experiment 2, the findings of Experiment 3 also suggest that the involvement of D1R and D2R in S2-S1 learning does not appear to differ as a function of the motivational significance of the context in which learning occurs, although this cannot be directly compared across the two experiments. The aim of Experiment 4 was to assess whether the detrimental effects of dopamine antagonism on S2-S1 conditioning are additive when D1- and D2-receptors are blocked together, as well as to provide a direct comparison of these effects when learning takes place in a motivationally neutral vs. motivationally attractive context.

Fifty-six male rats were randomly allocated to receive training in either a neutral or appetitive context. For the appetitive condition, rats were placed on a food restriction schedule during handling and context exposure occurred on Days 1 and 2. Day 2 involved exposure to food rewards throughout both sessions. Magazine entry data was unfortunately recorded incorrectly for eight of 24 rats in the appetitive condition. In the remaining 16, there was a significant increase in entries to the food magazine per min from the first to the second session of context exposure with food presentation [*t*-test; *t*_(15)_ = 4.695, *p* < 0.001, where mean (±SD) for session 1 = 8.400 (±3.493) and session 2 = 11.400 (±2.650)], which is at least suggestive that the manipulation was successful overall. For the Neutral condition, context exposure proceeded without any programmed events.

Rats in each condition were further randomly allocated to receive an injection of either saline (Groups Neutral-VEH and Appetitive-VEH) or α-flupenthixol (Groups Neutral-FLU and Appetitive-FLU) immediately prior to the SPC session. In this experiment, all rats received paired S2-S1 presentations during the SPC session on Day 3 and paired S1-shock presentations during the FOC session on Day 4. Context extinction was conducted on Day 5, and tests of S2 and S1 were carried out on Days 6 and 7, respectively. Final group sizes were as follows: Group Neutral-VEH, *n* = 16; Group Neutral-FLU, *n* = 16; Group Appetitive-VEH, *n* = 12; Group Appetitive-FLU, *n* = 12. The neutral condition was slightly overpowered as previous experiments suggest there is more variance in the data when animals are trained in a neutral vs. appetitive context.

**Figure 5:**
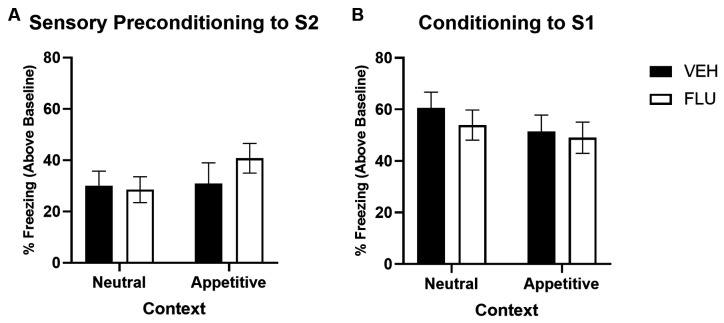
Sensory preconditioning under conditions of non-selective dopamine receptor antagonism when trained in a neutral vs. appetitive context. Panel **(A)** shows the average per cent time spent freezing in response to S2 (stimulus—baseline) on the test of sensory preconditioning for groups in the Neutral vs. Appetitive context condition. Panel **(B)** shows the average per cent time spent freezing to S1 (stimulus—baseline) on the test of first-order conditioning for groups in the Neutral vs. Appetitive context condition. Black bars indicate groups treated with saline vehicle (VEH; Neutral condition *n* = 16, Appetitive condition *n* = 12), and white bars indicate groups treated with α-flupenthixol (FLU; Neutral condition *n* = 16; Appetitive condition *n* = 12). Error bars represent ±SEM.

Figure 5A shows average levels of freezing in response to S2 at test (stimulus-baseline; averaged across eight S2 presentations) for groups that were training in a neutral vs. an appetitive context. No groups differences in levels of freezing to S2 were observed. Despite numerically higher levels of freezing in the α-flupenthixol-treated group trained in the appetitive context, a two-way univariate ANOVA revealed no main effect of Drug Type (VEH vs. FLU; *F* < 1) or Context (Neutral vs. Appetitive; *F*_(1,52)_ = 1.138, *p* = 0.291), or Drug Type by Context interaction (*F* < 1).

Figure 5B shows the average levels of freezing in response to S1 at the test for groups that were training in a neutral vs. an appetitive context. There was no evidence to suggest responses differed between groups, with a two-way univariate ANOVA revealing no significant effect of Drug Type (Saline vs. FLU; *F*_(1,52)_ = 1.292, *p* = 0.261), Context (Neutral vs. Appetitive; *F* < 1) or Drug Type by Context interaction (*F* < 1).

Thus, there is no evidence in this experiment to suggest that non-selective dopamine antagonism has any impact on learning S2-S1 associations, irrespective of the motivational significance of the context. This stands in explicit contrast to the findings of both Experiments 2 and 3 above, which demonstrate when either D1- or D2-receptors are blocked independently (in a neutral or appetitive context), S2-S1 learning is impaired.

## Discussion

In a preliminary set of experiments, we first established a sensory preconditioning procedure modelled on that used by Holmes and colleagues (e.g., Holmes et al., 2013). This procedure produced reliable sensory preconditioning that depended upon both the pairing of sensory cues S2 and S1 and the subsequent pairing of S1 with a mild foot-shock US. The magnitude of the sensory preconditioning effect was similar regardless of whether the conditioning context was neutral, or had previously been paired with a non-contingent appetitive outcome. These experiments provide a baseline for the investigation of the role of dopaminergic receptors in the development of sensory preconditioning.

We then examined the impact of dopamine D1R (SCH39166) and D2R (eticlopride) selective antagonists on the development of sensory preconditioning (in an affectively neutral context), when administered immediately prior to the critical SPC phase in which neutral cues S2 and S1 were paired. Primarily, we found that treatment with either the D1R antagonist or the D2R antagonist decreased the level of S2-S1 learning, as observed in the subsequent test session. In both instances, the degree of sensory preconditioning observed in drug-treated animals, as indexed by conditioned freezing to S2, was significantly lower than that seen in vehicle-injected control animals (and numerically lower than the levels of sensory preconditioning seen in the initial behavioural studies). Thus, both D1R and D2R activity appears important for learning an association between two innocuous sensory cues.

Experiment 2 also presented several other findings of note. In neither instance (D1R or D2R) did we find an effect of dopamine receptor antagonism on the level of conditioned freezing observed in the presence of the directly trained cue, S1; that is, conditioned freezing to this cue at test did not differ between drug-treated groups and vehicle-injected control groups, and reflected the relatively high level of freezing expected in a cue paired directly with mild foot-shock. This is unsurprising given the antagonists were administered only prior to S2-S1 pairings, but nevertheless provides evidence that the observed deficit in freezing to S2 was not secondary to some long-lasting impact of the drug-treatment on first-order conditioning.

We also did not observe any effect of the sex of the subjects on the level of sensory preconditioning or the level of first-order conditioning, and there were also no observed interactions between sex and the impact of dopaminergic receptor antagonism on these measures. Therefore, we did not find any evidence of differences in sensory preconditioning *per se*, nor in the relative importance of D1 and D2 dopamine receptors in sensory preconditioning, between male and female rats.

Thirdly, we did not find any evidence of a relationship between the dose of dopamine receptor antagonist administered and the degree of deficit observed in sensory preconditioning. Overall, low, middle and higher doses of either SCH39166 or eticlopride produced equivalent deficits in sensory preconditioning, and the magnitude of the deficit also did not significantly differ between D1R and D2R antagonism. The doses selected for SCH39166 and eticlopride were selected on the basis of several previous experiments, both in our lab and elsewhere, where dose-response effects have been observed (e.g., Nelson and Killcross, 2013; Hosking et al., 2015; Roughley, 2017; Roughley and Killcross, 2019) This suggests that dopamine D1R or D2R function is important in sensory preconditioning, but may fulfil a basic permissive role, rather than one governing level of stimulus-stimulus learning.

We would also note that sensory preconditioning was reduced by dopamine D1R or D2R antagonism to levels numerically similar to those seen in Groups P-UP and UP-P in Experiment 1 (though these cannot be directly compared). As such, it appears that the levels of sensory preconditioning observed following D1R and D2R antagonism are reduced to levels seen in groups in which S2 and S1 pairings (or S1-US pairings) had not occurred. Whilst this does not necessarily indicate that sensory preconditioning was completely abolished, it was reduced to a level which would not have been detectable compared to unpaired groups. As such, residual levels of freezing to S2 in the case of D1R and D2R antagonist-treated groups could well be unrelated to the formation of S2-S1 associations and may be due to non-associative processes such as stimulus generalisation at test (between S1 and S2), or cue-related priming of residual contextual fear. However, further experiments with appropriate within-experiment controls would be needed to fully explore these possibilities.

In a third experiment, we examined the impact of D1R or D2R antagonism on the formation of S2-S1 associations in a sensory preconditioning study conducted in a context which had been rendered affectively appetitive by prior presentations of food reward in that context. We did this to examine a number of issues. First, whilst we found no evidence for differential involvement of D1R and D2R in sensory preconditioning in our initial study, previous work has suggested that D1- and D2-related systems may be involved in dissociable aspects of learning about rewards; for example, it has been proposed that D1R systems may be more selectively involved in the encoding of prediction error in learning, whereas D2R systems may be more involved in the assignment of motivational significance to cues paired with reward (Roughley and Killcross, 2019). Second, previous work (Holmes et al., 2013, 2018; Holmes and Westbrook, 2017) has indicated that the neural bases of sensory preconditioning may vary depending on the motivational significance of the environment in which neutral S2-S1 pairings occur. In short, however, we failed to observe any influence of the motivational status of the training context on the impairment in sensory preconditioning observed following dopamine receptor antagonism. Replicating the previous studies, both D1R and D2R antagonism produced significant deficits in freezing to S2 to at test, whilst no deficits were seen in freezing to the directly trained cue S1.

In the final experiment, we sought to extend these findings in two ways. First, we sought to examine whether deficits in sensory preconditioning following D1R or D2R blockade were enhanced when both D1R and D2R antagonism was imposed simultaneously, following treatment with the non-selective dopamine D1R/D2R antagonist α-flupenthixol. Second, we sought to further examine the potential that the motivational status of the learning environment might have an impact under this increased breadth of dopamine receptor antagonism. Hence, we examined the impact of treatment with α-flupenthixol prior to sensory preconditioning S2-S1 pairings in either a neutral or appetitive learning environment. Surprisingly, though the dose of α-flupenthixol used was known to be behaviourally effective (see below), we found that combined D1R/D2R antagonism failed to produce deficits in freezing to S2 (or S1) in either a neutral or appetitive learning environment. Potential explanations for this finding are discussed below, including the possibility that at these doses the different antagonists have distinct loci of action, or that perhaps performance relies less on absolute levels of activity at D1/D2 receptors, but rather the balance of activity between the two.

In summary, we employed a procedure for sensory preconditioning in which we demonstrated that the pairing of neutral cues S2 and S1 is required for the development of conditioned responding to S2 following S1-US pairings. This sensory preconditioning effect was markedly reduced following administration of dopamine D1R or D2R antagonists prior to S2-S1 pairings. This effect did not vary across male and female rats and was uninfluenced by the motivational status of the training environment. Unexpectedly, these deficits in sensory preconditioning were not seen following administration of the D1/D2R antagonist α-flupenthixol at an otherwise effective dose.

The overall aim of this set of experiments was to use the sensory preconditioning protocol to examine dopaminergic involvement in associative learning processes (e.g., prediction error) whilst controlling for the motivational processes that typically accompany learning in a reward setting (and are also believed to be dopamine-dependent). Broadly speaking, these data confirm and extend previous findings indicating a role for dopamine systems that goes beyond reward prediction error (Sharpe et al., 2017; Gardner et al., 2018). Whilst the evidence for a role for dopamine in reward prediction error is strong, there is also mounting evidence that the role of dopamine extends to learning about stimulus-related prediction errors, as well as aversive events. This adds to the basic initial observation that midbrain dopamine neurons fire strongly to unanticipated sensory cues (Ljungberg et al., 1992; Schultz and Dickinson, 2000). For example, Sharpe et al. (2017) have demonstrated that dopamine transients are sufficient and necessary for the formation of stimulus-stimulus associations, using a version of a sensory preconditioning task. This has led some to suggest a role for dopamine in subserving a generalised prediction error term that signals errors in both sensory and reward predictions (Suri, 2001; Gershman, 2017; Gardner et al., 2018).

Our findings are in line with these models in demonstrating a role for dopaminergic systems in stimulus-stimulus learning. However, rather than concentrating on the role of dopaminergic signalling of error terms, our experiments also demonstrate the downstream role of dopamine binding to D1R and D2R. Our initial experiments suggest that antagonism of either D1R or D2R is capable of attenuating the formation of S2-S1 associations. This constitutes an important next step in identifying the neural pathways recruited for this learning and thereby being able to determine commonalities and differences with reward-based learning that can inform our understanding of the basic mechanisms underpinning both. For example, the absence of any dose-response curve in the effects of systemic D1R or D2R antagonism perhaps suggests a permissive, rather than graded, role—a possibility that invites further experimentation in the future. Although higher systemic doses of these antagonists have general motor and arousal effects that would preclude a clear interpretation of findings, other approaches are possible, including localised receptor inactivation by the antagonist and chemogenetic or optogenetic inhibition.

That the independent role of D1R and D2R appear similar in this task is in contrast to some findings where opposing effects of D1R and D2R manipulation are seen (Eyny and Horvitz, 2003; Yue et al., 2004; Nelson and Killcross, 2013), but there is also substantial evidence where the impact of D1 and D2 systems has been shown to be complementary (Ikemoto et al., 1997; Smith et al., 1997; Capper-Loup et al., 2002; Iordanova et al., 2006; Cerri et al., 2014; Perreault et al., 2014). The effect we have reported here falls into this latter category, and also provides a fruitful opportunity for further investigation. For example, our findings may suggest an impact of these systemic treatments in areas of the brain (such as the nucleus accumbens) where D1R and D2R systems are less clearly in opposition than has been reported in other areas (e.g., dorsal striatum; Gerfen and Surmeier, 2011; Kravitz et al., 2012; Kupchik et al., 2015). Future experiments employing anatomically specific dopamine receptor manipulations to identify the neural circuitry involved will be important for describing the precise nature of the functions performed by D1R and D2R in this context.

A further extension of previous work is the present finding that the importance of D1R and D2R in sensory preconditioning does not appear to differ as a function of the motivational status of the learning environment. One limitation of investigating dopamine’s role in the processes of learning associative relationships has been the fact that this research has primarily been conducted using reward-based learning procedures in which the learning context is established as attractive prior to learning as a typical part of an experimental protocol (i.e., pre-training involves several sessions of exposure to reward in the training context to familiarise the animal with both before training begins). Although research has demonstrated that the motivational significance of the training environment can have a significant impact on the neural mechanisms recruited for learning (Holmes et al., 2013, 2018), this has not been explicitly examined or controlled for in the context of dopamine despite established effects of dopaminergic systems in appetitive contextual conditioning (Spyraki et al., 1982a,b; Beninger and Hahn, 1983; Spyraki et al., 1987). In doing so, the present findings further confirm not only that dopamine is important for learning relationships between neutral stimuli, but that this is equally true in a familiar but neutral environment as it is in an attractive one.

Our assumption that the training context was rendered attractive is evidenced by the observation that entries into the food magazine increased with exposure to food within the context. However, one could argue that this was not a particularly strong manipulation and that it only addresses the appetitive, not aversive, domain. Whilst this is true, and more powerful (appetitive or aversive) manipulations could be employed, the present manipulation was designed to be in keeping with procedures typically employed in experiments of reward-based learning.

Intriguingly, despite having found clear evidence for independent effects of D1R and D2R antagonism across two experiments, we failed to observe any impact of combined D1/D2R antagonism on sensory preconditioning. Firstly, this is unlikely to be because the antagonist was simply without effect. The dose of the α-flupenthixol (0.5 mg/kg) was the same as (or higher than) doses used in our lab and elsewhere, which have established impacts on learning and performance (Killcross et al., 1994; Dickinson et al., 2000; Dunn and Killcross, 2007). Further, we used the same dose from the same batch of α-flupenthixol to examine the impact of D1R/D2R antagonism on appetitive Pavlovian conditioned responding (something we have replicated several times in our lab). Here we found that 0.5 mg/kg α-flupenthixol, administered prior to an appetitive conditioning session in a manner identical to the administration prior to S2-S1 pairings in Experiment 4, drastically reduced the level of appetitive conditioned responding relative to control animals [magazine entries per min; *t*-test, *t*_(14)_ = 7.210, *p* < 0.001, where mean (±SD) for saline-treated rats = 11.767 (±1.309) and α-flupenthixol-treated rats = 4.213 (±2.660)]. There are also numerous examples (again, from our lab and elsewhere) where this dose of α-flupenthixol has been shown to be capable of mimicking the impact of more selective dopamine D1R and D2R antagonists at the doses used in our earlier experiments (e.g., Roughley and Killcross, 2019).

Accordingly, we have good reason, both within this series of studies and from others, to believe this dose of α-flupenthixol would have been effective. And whilst we would note caution around drawing strong conclusions on the basis of a null result finding, the failure to find an effect of combined D1/D2 antagonism does stand in contrast to the findings of the previous experiments, following the same methodology, in which clear effects of D1R and D2R antagonism alone were observed. Future studies may benefit from a within-experiment comparison of the effects of selective vs. non-selective D1R/D2R antagonists. Nevertheless, if we take this result at face value, there would be few studies that fail to find similar effects of combined D1/D2R antagonism when both D1R and D2R antagonism has been shown to be effective. Although speculative, there are some potential routes for future investigation outlined below.

One possibility might lie in the locus of action of the selective D1R and D2R antagonists, and the combined antagonist, particularly at the doses used in these experiments. A more discriminatory impact on inhibitory D2 autoreceptors (as opposed to post-synaptic D2R) could, for example, play a role if these proved to be differentially sensitive to selective and non-selective dopamine receptor antagonists. Similarly, given what we know about other neural substrates of sensory preconditioning (Ward-Robinson et al., 2001; Coutureau et al., 2002; Holmes et al., 2018; Fournier et al., 2021; Kahnt and Schoenbaum, 2021), as well as the different roles of dopamine in striatal regions (e.g., Young et al., 1998; Li and McNally, 2015; Yee et al., 2020), there could be a more complicated interplay of dopaminergic involvement than can be teased out with systemic drug administration studies. Accordingly, central administration studies will be needed to help clarify this in the future.

Given the potential that functioning of D1R and D2R systems in attenuating sensory preconditioning appears to operate as a logical NAND gate, then another possibility to explain the differential impact of selective vs. non-selective dopamine antagonists is that it might be the relative balance of D1R and D2R activity that is critical. That is, it may be that blocking either D1R or D2R activity and disrupting the relative balance between the two results in impaired sensory preconditioning. In contrast, blocking both D1R and D2R together reduces dopamine signalling overall, but preserves the relative balance of D1R and D2R activity and allows sensory preconditioning to proceed unimpaired. This notion is not without precedent. For example, Furlong et al. (2017) demonstrated that methamphetamine sensitisation reduced activity in D1R-expressing direct pathway neurons in the dorsomedial striatum (relative to D2R-expressing indirect pathway neurons), and that behavioural deficits observed following this impact could be reduced by pre-test administration of the adenosine 2A receptor antagonist ZM241385 into the dorsomedial striatum to reduce activity in D2R-expressing neurons. They hypothesised that this additional reduction in activity restored the balance of D1- and D2-related activity in the striatum and hence restored normal behavioural control. It is also the case that the potential heterogeneity of dopamine responses has been highlighted by recent theoretical models that seek to broaden the role of dopamine beyond reward prediction error (Suri, 2001; Gardner et al., 2018). Additional experiments, for example investigating sensory preconditioning under conditions of D1R and D2R agonism, would be needed to address this possibility.

In summary, both dopamine D1R and D2R activity appear to be independently important for sensory preconditioning in a manner which is also independent of the motivational status of the learning environment. This confirms and extends previous findings indicating a role for dopamine signalling beyond reward prediction error, and lays the groundwork for further investigation of the downstream mechanisms supporting this function in dopamine systems. Moreover, these findings underscore the utility of the sensory preconditioning procedure as a contrast to more typical reward-based learning procedures in further delineating the range of dopaminergic function in learning. However, the potential that combined dopamine D1R and D2R blockade does not impact sensory preconditioning further highlights the complexity of signalling in dopaminergic systems in relation to learning. In decoding this complexity, future studies are well-placed to employ neuroanatomically targeted approaches to identify the brain regions in which D1R and D2R activity exerts an influence in sensory preconditioning and undertake more direct and controlled manipulations, for example in the balance of activity in D1R- and D2R-expressing neural populations. Furthering our understanding of dopamine function in this manner has important clinical implications for our understanding of psychological disorders involving dysfunctional associative and motivational processes, such as in addiction, schizophrenia, depression, and ADHD.

## Data Availability Statement

The raw data supporting the conclusions of this article will be made available by the authors, without undue reservation.

## Ethics Statement

The animal study was reviewed and approved by UNSW Animal Care and Ethics Committee.

## Author Contributions

SR and SK conceived and designed the experiments. SR and AM collected and analysed the data. SR and SK interpreted the data and wrote the manuscript, with input from AM. All authors contributed to the article and approved the submitted version.

## Conflict of Interest

The authors declare that the research was conducted in the absence of any commercial or financial relationships that could be construed as a potential conflict of interest.

## Publisher’s Note

All claims expressed in this article are solely those of the authors and do not necessarily represent those of their affiliated organizations, or those of the publisher, the editors and the reviewers. Any product that may be evaluated in this article, or claim that may be made by its manufacturer, is not guaranteed or endorsed by the publisher.
